# Supplementary material for: Value of Cellular Components and Focal Dedifferentiation to Predict the Risk of Metastasis in a Benign-Appearing Extra-Meningeal Solitary Fibrous Tumor: An Original Series from a Tertiary Sarcoma Center
Source: Cancers (Basel). 2023 Feb 24;15(5):1441. doi: 10.3390/cancers15051441 (PMC10000949; doi:10.3390/cancers15051441)
Supplement: Supplementary file 1 [file cancers-15-01441-s001.zip › cancers-2143039-supplementary.pdf]

A 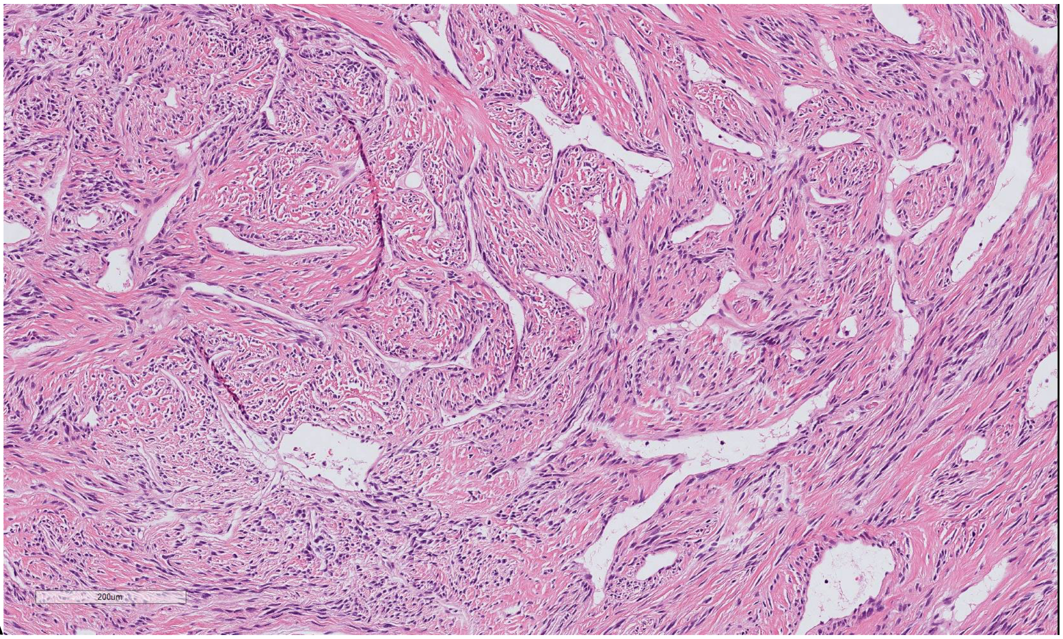

Figure 2A: Classic hyalinized solitary fibrous tumor

B 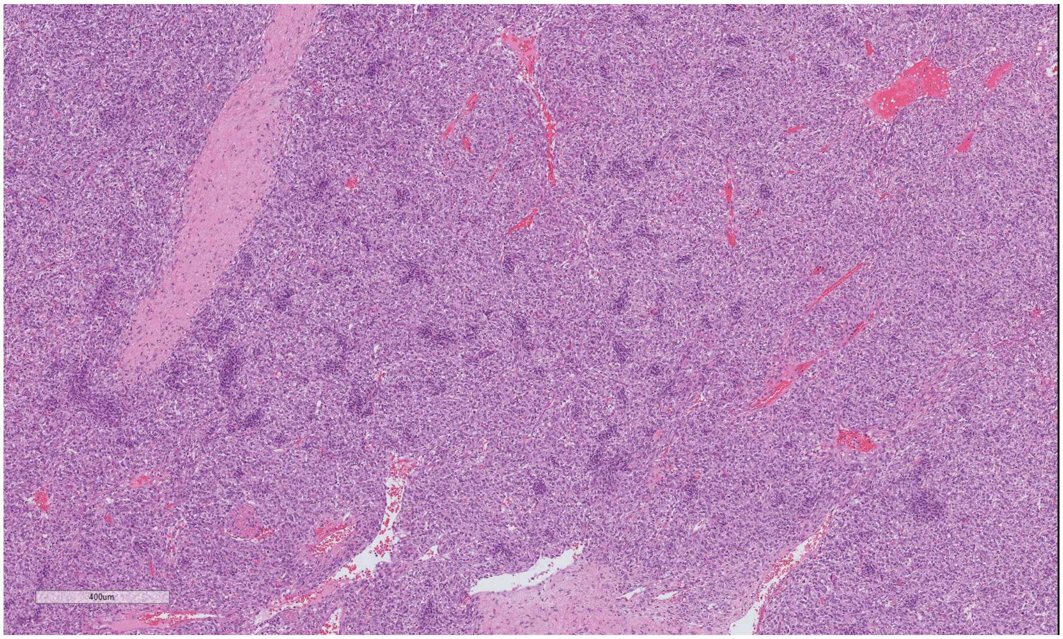

Figure 2B: Cellular variant of the tumor. The cellular SFT is characterized by a tightly packed proliferation of ovoid to spindle cells arranged around conspicuous vessels and scant stromal components.

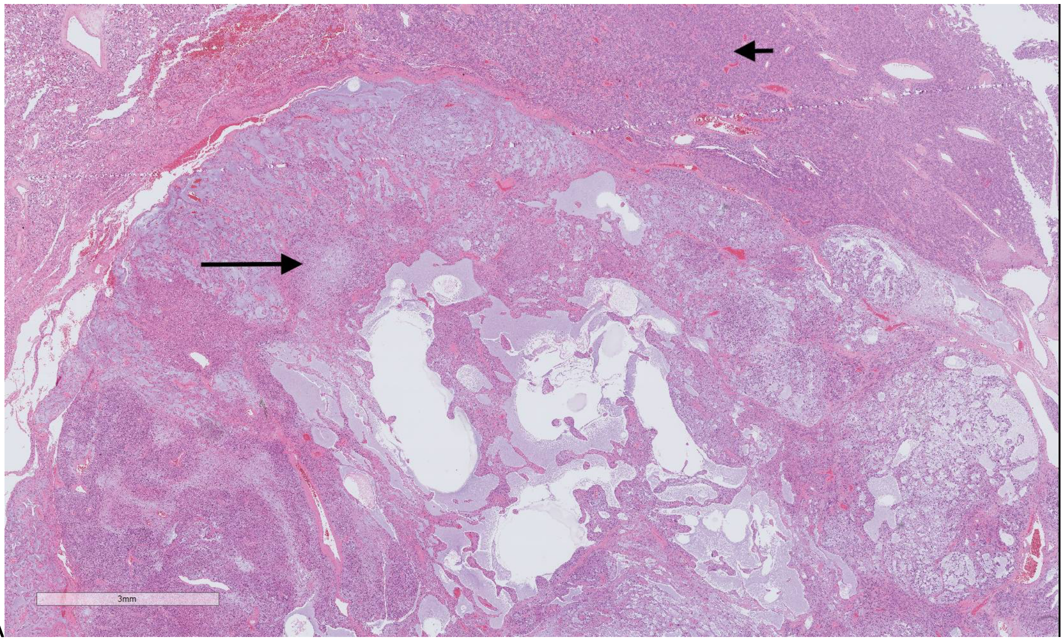

A

Figure 6. Dedifferentiated Solitary Fibrous Tumor H&E staining. Two distinct areas of typical solitary fibrous tumor (upper part/short arrow) and high-grade pleomorphic sarcoma (lower part/long arrow).
